# On-treatment change in bone turnover markers predicts 2-year bone mineral density after sequential therapy following romosozumab: a real-world cohort study

**DOI:** 10.3389/fendo.2026.1858266

**Published:** 2026-06-17

**Authors:** Ryo Nakano, Ayumi Ichisawa, Kenya Saruta, Masakazu Kogawa, Akira Fukuda

**Affiliations:** Department of Orthopedic Surgery, Mutsu General Hospital, Shimokita Medical Center Association, Mutsu, Japan

**Keywords:** bone mineral density, bone turnover markers, osteoporosis, P1NP, real-world study, romosozumab, sequential therapy, TRACP-5b

## Abstract

**Background:**

Romosozumab significantly increases bone mineral density (BMD); however, identifying patients who will achieve the greatest long-term BMD gains after sequential therapy remains challenging. BTMs decrease markedly during romosozumab treatment; however, whether the magnitude of this on-treatment change predicts 2-year cumulative BMD has not been established.

**Methods:**

In this retrospective single-center cohort study (n=315; April 2019–April 2025; ethics approval RO7-5), we analyzed 129 patients with complete on-treatment BTM pairs and 24-month BMD data. On-treatment changes in procollagen type I N-terminal propeptide (ΔP1NP) and tartrate-resistant acid phosphatase 5b (ΔTRACP-5b) were calculated as the difference between post-treatment and pre-treatment values. The primary outcome was the cumulative percentage change in lumbar spine BMD (LS-BMD) from baseline to 24 months. Spearman’s rank correlations and multiple linear regression adjusting for age, baseline LS-BMD, prior treatment, sequential therapy type, and 12-month BMD change were performed. ROC analysis defined an optimal ΔP1NP cutoff for predicting a good response, and subgroup analyses examined the predictive value by sequential therapy type and prior treatment status.

**Results:**

ΔP1NP was significantly correlated with the 24-month cumulative LS-BMD change (ρ=−0.375, p<0.0001, n=129), and a greater decline in P1NP during romosozumab treatment predicted greater cumulative BMD gain. ΔTRACP-5b showed a similar association (ρ=−0.348, p=0.0001, n=114). In multiple linear regression adjusted for all covariates, ΔP1NP remained an independent predictor (β=−0.061, p=0.010; model R²=0.796, n=113). The optimal ΔP1NP cutoff was −40.3 μg/L (AUC = 0.761; sensitivity=0.545, specificity=0.854). Patients in the highest ΔP1NP decline tertile (T1) achieved 23.5% cumulative LS-BMD gain versus 12.3% in the lowest tertile (T3; p=0.0003). This association was replicated in the denosumab sequential therapy subgroup (ρ=−0.354, p=0.0010, n=84). The predictive value was stronger in treatment-naïve patients (ρ=−0.359, p=0.0021) than in previously treated patients (ρ=−0.207, p=0.120; interaction p=0.024).

**Conclusion:**

On-treatment ΔP1NP is an independent predictor of 2-year cumulative BMD after sequential therapy following romosozumab, with the greatest predictive value in treatment-naïve patients. These findings support the clinical utility of BTM monitoring during romosozumab treatment; however, given the single-center retrospective design, predominantly female Japanese population (96.9%), and unvalidated ROC cutoff, prospective multicenter validation is required before clinical implementation.

## Introduction

Romosozumab, an anti-sclerostin monoclonal antibody, exerts a unique dual effect by promoting bone formation and simultaneously suppressing bone resorption (1, 2). The pivotal FRAME and ARCH trials demonstrated significant reductions in vertebral and hip fractures (3, 4), establishing romosozumab as a first-line anabolic option for patients at a very high fracture risk. Following a 12-month romosozumab treatment course, sequential antiresorptive therapy, most commonly denosumab or bisphosphonates, is required to consolidate and maintain the BMD gains achieved (5).

Several studies have identified baseline bone turnover markers (BTMs) as predictors of the response to romosozumab. Higher baseline P1NP and TRACP-5b levels, reflecting greater pre-existing bone turnover, are associated with larger BMD gains during treatment (6–8). A combined classification of both markers has been shown to identify patients with differential 12-month BMD responses (9). Tominaga et al. identified a P1NP cutoff of approximately 50 ng/mL at 1 month as a predictor of non-response to romosozumab at the hip (8). Low baseline P1NP (<50 μg/L) has been identified as a biomarker for residual fracture risk after romosozumab treatment completion (10). Nonetheless, a critical clinical gap remains: whether the magnitude of the on-treatment BTM change, which captures the pharmacodynamic response to romosozumab itself, predicts long-term BMD beyond the end of the treatment course.

During romosozumab treatment, P1NP first rises transiently (reflecting the anabolic effect) and then declines, whereas TRACP-5b decreases consistently from early in treatment (11). Although published studies have examined on-treatment BTM changes as descriptive outcomes, no study to date has tested whether the magnitude of on-treatment ΔP1NP or ΔTRACP-5b at treatment completion independently predicts the cumulative 2-year BMD change achieved after sequential therapy administration. Critically, in our prior work (9), baseline P1NP and TRACP-5b as continuous variables did not retain independent significance in multivariate regression (P1NP: β=0.011, p=0.375; TRACP-5b: β=−0.003, p=0.645), suggesting that the dynamic change during treatment may capture information beyond the static pretreatment value.

Therefore, we investigated whether on-treatment ΔP1NP and ΔTRACP-5b predict 2-year cumulative LS-BMD change in a real-world cohort of 315 patients treated with romosozumab over a 6-year period. We further evaluated the optimal ΔP1NP cutoff using ROC analysis, performed subgroup analyses by sequential therapy type, and tested for effect modification according to prior treatment status.

## Materials and methods

### Study design and participants

This single-center retrospective cohort study was conducted at the Mutsu General Hospital, a regional core hospital in Aomori Prefecture, northern Japan. All consecutive patients who received 12 monthly subcutaneous injections of 210 mg romosozumab for high-risk osteoporosis between April 2019 and April 2025 were enrolled (n=315). The study population predominantly comprised patients with primary osteoporosis. The systematic exclusion of secondary osteoporosis etiologies (e.g., glucocorticoid-induced osteoporosis, hyperparathyroidism, and malignancy-associated bone disease) was not mandated by the protocol; however, treating physicians applied standard clinical judgment in selecting romosozumab candidates. Diabetes mellitus was present in 19 patients (6.0%), and all patients met the Japanese insurance eligibility criteria for romosozumab (high-fracture-risk osteoporosis). This study was approved by the Institutional Review Board of Shimokita Medical Center Association (approval number RO7-5), and informed consent was obtained from all participants. This study was conducted in accordance with the Declaration of Helsinki.

**Figure 1:**
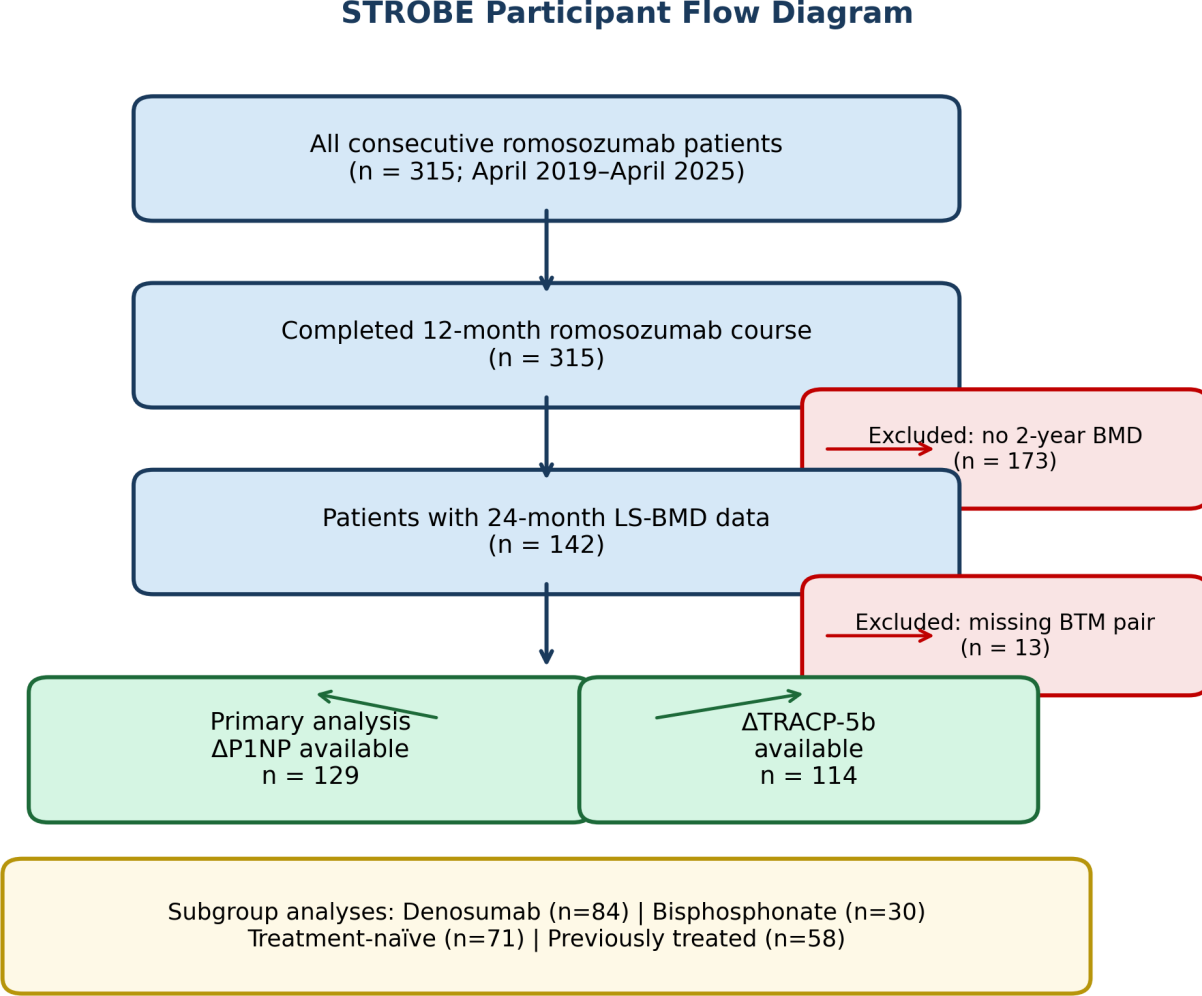
STROBE participant flow diagram. Of the 315 patients enrolled, 129 had complete data for the primary ΔP1NP analysis (paired BTM measurements and 24-month LS-BMD). Alt text: Flow diagram showing patient inclusion from 315 enrolled to 129 in the primary analysis, with reasons for exclusion.

The inclusion criteria for the primary analysis were as follows: (1) complete paired BTM measurements (P1NP and/or TRACP-5b at baseline and approximately 1 month after the 12th romosozumab dose) and (2) available 24-month LS-BMD data. Patients without 24-month BMD data or missing BTM pairs were excluded from the primary analysis (**Figure 1**). A total of 129 patients met the criteria for the ΔP1NP analysis, and 114 met the criteria for the ΔTRACP-5b analysis.

### Measurements

BMD was measured using dual-energy X-ray absorptiometry (DXA; Hologic Horizon W, Hologic Inc., Marlborough, MA, USA) at the lumbar spine (L1–L4) and total hip at baseline, 12 months (end of romosozumab), and 24 months (12 months after sequential therapy initiation). BMD values were expressed as a percentage of the young adult mean (%YAM), the standard Japanese reporting metric (12). In Japan, %YAM (where 100% represents the mean BMD of healthy adults aged 20–44 years) is the standard clinical reporting metric and is used to define osteoporosis (<70% YAM) and eligibility for pharmacotherapy. The corresponding approximate T-scores were −2.8 at the lumbar spine and −2.4 at the total hip in our cohort.

P1NP was measured using an electrochemiluminescence immunoassay (ECLIA; Roche Diagnostics, reference range 26.4–98.2 ng/mL) and TRACP-5b using an enzyme immunoassay (EIA; DS Pharma, reference range 120–420 mU/dL) at baseline (before the first romosozumab dose) and approximately 1 month after the final dose. On-treatment changes were calculated as ΔP1NP = P1NP(post) − P1NP(pre) and ΔTRACP-5b = TRACP-5b(post) − TRACP-5b(pre). Negative values indicate marker suppression.

### Outcomes

The primary outcome was the cumulative percentage change in LS-BMD from baseline to 24 months: [(LS-BMD at 24 months − LS-BMD at baseline)/LS-BMD at baseline] × 100. For ROC analyses, a “good responder” was defined as achieving a cumulative LS-BMD gain ≥75th percentile of the cohort (≥24.56%). This threshold was selected to identify the upper quartile of treatment responders, a convention used in previous BTM-BMD prediction studies (8). To assess the robustness of this definition, sensitivity analyses using alternative thresholds (≥50th percentile, ≥15%, and ≥3% [least significant change]) were performed, the and results are presented in **Supplementary Table 2**.

### Statistical analysis

Spearman’s rank correlation coefficients (ρ) were calculated between ΔP1NP/ΔTRACP-5b and the 24-month cumulative LS-BMD change. Multiple linear regression with 24-month cumulative LS-BMD as the dependent variable was performed, adjusting for age, baseline LS-BMD, prior treatment history (binary: naïve vs. previously treated), sequential therapy type (denosumab vs. bisphosphonate), and 12-month LS-BMD percentage change (a proxy for the romosozumab treatment effect). Standardized beta coefficients were calculated to compare the predictive value of ΔP1NP (absolute) versus ΔP1NP (percentage change); model fit was compared using the Akaike Information Criterion (AIC) and receiver operating characteristic (ROC) area under the curve (AUC).

Receiver operating characteristic (ROC) analysis was used to identify the optimal ΔP1NP cutoff for predicting a good response, with the Youden index (sensitivity + specificity − 1) used to identify the optimal threshold. ΔP1NP tertile analyses used the Mann-Whitney U test for comparisons between extreme tertiles (T1 vs. T3). Effect modification by prior treatment status was tested using a ΔP1NP × naïve interaction term in the regression model. All analyses were performed using Python 3.11 with the following libraries: SciPy 1.11, Statsmodels 0.14, Scikit-learn 1.3, and Pandas 2.1. ROC analysis used sklearn.metrics.roc_curve with the Youden index (sensitivity + specificity − 1) to identify the optimal threshold. Tertiles were defined as the 33.3rd and 66.7th percentiles of the ΔP1NP distributions. The interaction term between ΔP1NP and prior treatment status (binary: naïve vs. previously treated) was tested by adding a multiplicative term (ΔP1NP × naïve) to the base-regression model. All codes are available upon reasonable request from the corresponding author. A two-sided p<0.05 was considered statistically significant.

## Results

### Patient characteristics

Of the 315 enrolled patients, 129 had complete data for the ΔP1NP primary analysis (**Table 1**). The cohort was predominantly female (96.9%) and had a mean age of 76.4 years. Treatment-naïve patients comprised 55.0% (n=71), and 65.1% received denosumab as sequential therapy. The median cumulative LS-BMD gain at 24 months was 14.5% (IQR, 7.5–24.0%). Baseline characteristics did not differ significantly between patients with and without 24-month data, confirming a minimal attrition bias. The vast majority of patients were considered to have primary osteoporosis based on clinical assessment, and 19 (14.7%) had concomitant diabetes mellitus. According to the Japanese insurance eligibility criteria, the distribution of high fracture-risk definitions was as follows: existing fragility fractures ≥2 (48.8%), multiple criteria (25.6%), BMD ≤70% of YAM with fragility fracture (16.3%), LS-BMD ≤60% of YAM (7.8%), and severe vertebral compression (1.6%). Additional baseline biochemical parameters are shown in **Supplementary Table 1**.

**Table 1:** Baseline characteristics of the study population (n=129).

Variable	All patients (n=129)	Treatment-naïve (n=71)	Previously treated (n=58)
Age (years), mean ± SD	76.4 ± 8.1	75.9 ± 8.3	77.1 ± 7.9
Female, n (%)	125 (96.9)	69 (97.2)	56 (96.6)
Baseline LS-BMD (%YAM), mean ± SD	70.4 ± 12.6	68.1 ± 13.0	73.2 ± 11.6
Baseline P1NP (μg/L), mean ± SD	68.2 ± 47.8	82.4 ± 53.1	50.9 ± 34.2
Baseline TRACP-5b (mU/dL), mean ± SD	490.2 ± 228.4	568.4 ± 241.2	393.6 ± 188.3
ΔP1NP (μg/L), mean ± SD	−11.9 ± 45.2	−18.4 ± 48.9	−4.0 ± 39.1
Sequential therapy			
Denosumab + VitD, n (%)	84 (65.1)	46 (64.8)	38 (65.5)
Bisphosphonate + VitD, n (%)	30 (23.3)	18 (25.4)	12 (20.7)
Other/VitD alone, n (%)	15 (11.6)	7 (9.9)	8 (13.8)
24-month cumulative LS-BMD change (%), median [IQR]	14.5 [7.5–24.0]	17.2 [9.1–27.3]	11.8 [5.6–19.4]

Data are presented as mean ± SD, n (%), or median [IQR]. LS-BMD, lumbar spine bone mineral density; %YAM, percentage of young adult mean; IQR, interquartile range; VitD, active vitamin D.

### On-treatment BTM changes

ΔP1NP showed a wide range (−146.6 to +133.1 μg/L; mean −11.9 ± 45.2 μg/L), and approximately 62% of patients showed a net decline. ΔTRACP-5b was consistently negative (mean −87.4 ± 142.3 mU/dL), reflecting the anti-resorptive effect of romosozumab.

### Primary analysis: ΔP1NP and 24-month LS-BMD

**Figure 2:**
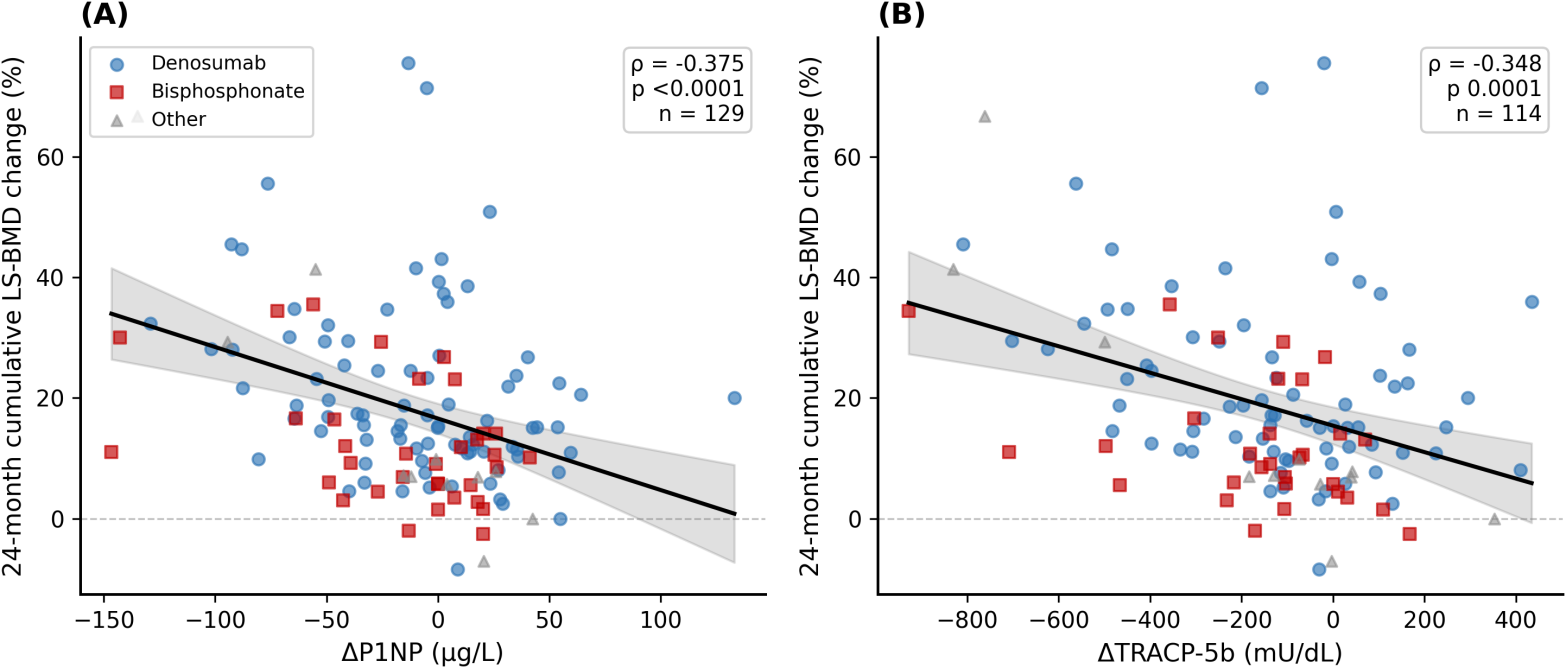
Scatter plots of on-treatment BTM changes versus 24-month cumulative LS-BMD change. **(A)** ΔP1NP vs. cumulative LS-BMD % (n=129; ρ=−0.375, p<0.0001). **(B)** ΔTRACP-5b vs. cumulative LS-BMD % (n=114; ρ=−0.348, p=0.0001). The points are colored according to the sequential therapy type (denosumab: blue; bisphosphonate: red). The regression lines and 95% confidence intervals are shown. Alt text: Two scatter plots showing inverse correlations between on-treatment BTM decline and 24-month cumulative BMD gain.

ΔP1NP was significantly inversely correlated with the 24-month cumulative LS-BMD change (Spearman ρ=−0.375, p<0.0001, n=129; **Figure 2A**). Greater suppression of P1NP during romosozumab treatment was associated with greater cumulative BMD gain at 24 months. ΔTRACP-5b showed a similar significant association (ρ=−0.348, p=0.0001, n=114; **Figure 2B**).

In multiple linear regression adjusted for age, baseline LS-BMD, prior treatment status, sequential therapy type, and 12-month LS-BMD change, ΔP1NP remained a significant independent predictor of 24-month cumulative LS-BMD (β=−0.061, p=0.010; **Table 2**). The full model explained 79.6% of the variance in 24-month BMD (R²=0.796, n=113), with the 12-month LS-BMD change emerging as the dominant covariate.

**Table 2:** Multiple linear regression: independent predictors of the 24-month cumulative LS-BMD change (n=113).

Variable	β coefficient	95% CI	Standardized β	p-value
ΔP1NP (μg/L)	−0.061	−0.108, −0.015	−2.384	0.010
12-month LS-BMD change (%)	0.884	0.742, 1.026	0.812	<0.001
Baseline LS-BMD (%YAM)	−0.142	−0.248, −0.036	−0.178	0.009
Age (years)	−0.098	−0.241, 0.045	−0.074	0.178
Prior treatment (yes vs. no)	−1.842	−4.102, 0.418	−0.098	0.110
Sequential therapy (denosumab vs. BP)	2.104	−0.224, 4.432	0.106	0.076
Model R² = 0.796; Adjusted R² = 0.782; n = 113				

LS-BMD, lumbar spine bone mineral density; %YAM, percentage of young adult mean; BP, bisphosphonate; CI, confidence interval.

### ROC analysis and optimal ΔP1NP cutoff

**Figure 3:**
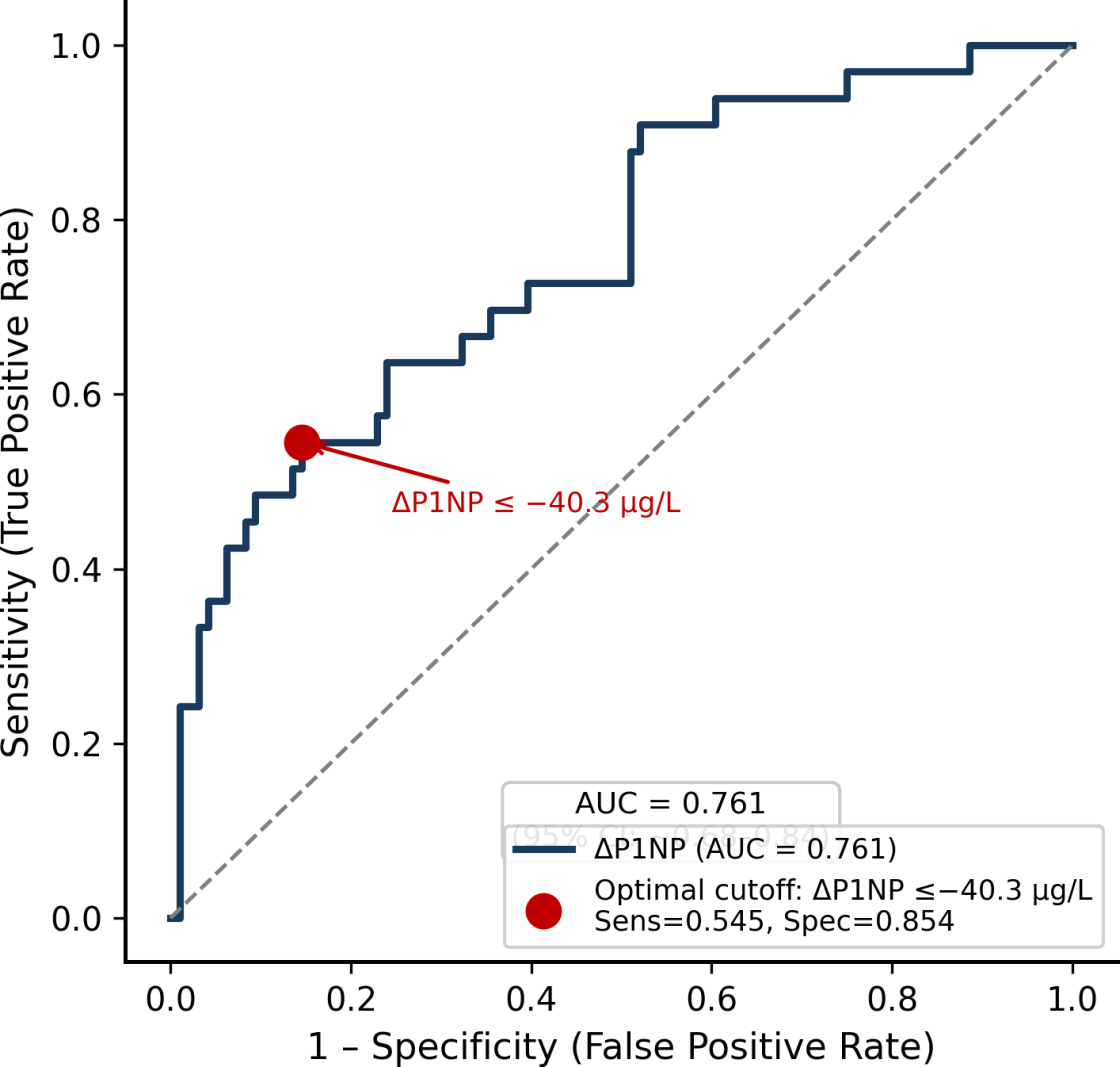
Receiver operating characteristic (ROC) curve for ΔP1NP predicting good responders (cumulative LS-BMD ≥24.56%). AUC = 0.761. The optimal Youden index cutoff (ΔP1NP ≤−40.3 μg/L) is marked with a filled circle (sensitivity 0.545, specificity 0.854). The dashed line represents a reference diagonal. Alt text: ROC curve with AUC 0.761 and optimal cutoff point.

ROC analysis for predicting a good response (cumulative LS-BMD ≥24.56%) yielded an AUC of 0.761 for ΔP1NP (absolute value; **Figure 3**). The optimal Youden index cutoff was ΔP1NP ≤−40.3 μg/L (sensitivity 0.545, specificity 0.854, Youden index 0.400). In comparison, the ΔP1NP percentage change showed an AUC of 0.728 (optimal cutoff −18.2%). Standardized regression confirmed the superiority of the absolute change (β=−2.384, p=0.0018, AIC = 892.6) over the percentage change (β=−1.126, p=0.151, AIC = 901.3).

### Tertile and dichotomous analyses

**Figure 4:**
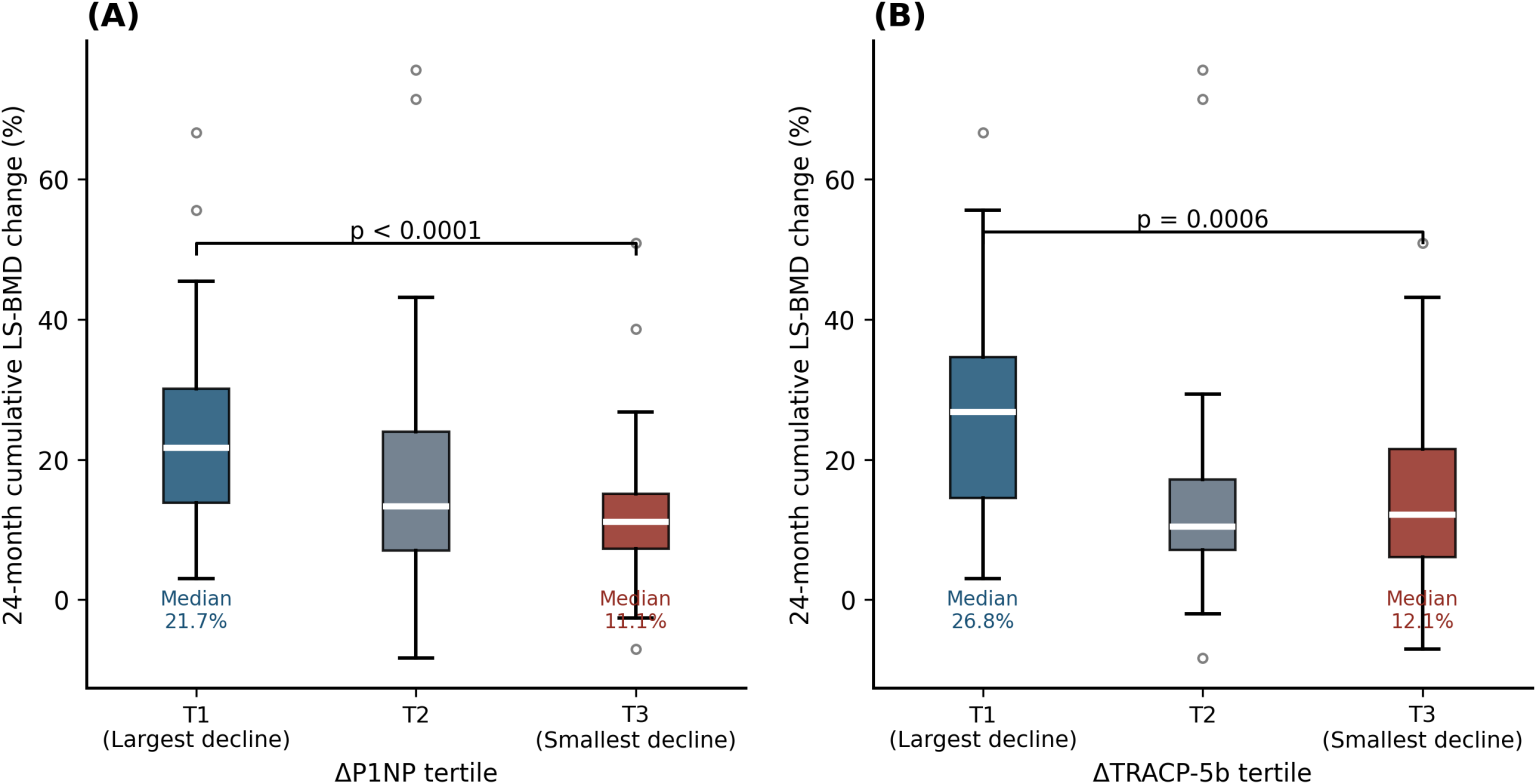
Box plots of the 24-month cumulative LS-BMD change by on-treatment BTM decline tertile. **(A)** ΔP1NP tertiles: T1 (largest decline, n=43) 23.5% vs. T3 (smallest decline, n=43) 12.3% (p=0.0003). **(B)** ΔTRACP-5b tertiles: T1 26.1% vs. T3 15.1% (p=0.0001). Boxes show the IQR, horizontal lines show the medians, whiskers extend to 1.5× IQR, and individual points show outliers. Alt text: Two box plots showing higher cumulative BMD gains in patients with greater on-treatment BTM suppression.

ΔP1NP tertile analysis demonstrated a significant gradient in the 24-month cumulative BMD gain: T1 (largest decline) 23.5% vs. T3 (smallest decline) 12.3% (Mann-Whitney U, p=0.0003; **Figure 4A**). Similarly, ΔTRACP-5b tertile analysis showed T1 26.1% vs. T3 15.1% (p=0.0001; **Figure 4B**). Among patients showing any net decrease in P1NP (n=80), the cumulative BMD gain was 21.7% versus 13.8% in those with a net increase (n=49, p=0.0016).

### Subgroup analyses by sequential therapy type

In the denosumab sequential therapy subgroup (n=84), ΔP1NP remained significantly correlated with the 24-month cumulative LS-BMD (ρ=−0.354, p=0.0010), confirming that the association is not confined to bisphosphonate users. In the bisphosphonate subgroup (n=30), a numerically similar correlation was observed (ρ=−0.350), but it did not reach statistical significance (p=0.058), likely reflecting insufficient statistical power. The ΔP1NP tertile effect was consistent within the denosumab group (T1 vs. T3 cumulative BMD: 25.8% vs. 14.2%, p=0.012; **Supplementary Table 1**).

### Effect modification by prior treatment status

The predictive value of ΔP1NP differed significantly between treatment-naïve and previously treated patients (interaction term β=−0.072, p=0.024; **Table 3**). In treatment-naïve patients (n=71), ΔP1NP was strongly associated with 24-month BMD (ρ=−0.359, p=0.0021), with the T1 tertile achieving 28.7% versus 15.4% cumulative gain in T3 (p=0.0013). In previously treated patients (n=58), the correlation was attenuated and non-significant (ρ=−0.207, p=0.120), and the tertile difference did not reach significance (T1: 19.2% vs. T3: 12.4%, p=0.199). ΔTRACP-5b showed a similar pattern (naïve: ρ=−0.407, p=0.0009; experienced: ρ=−0.144, p=0.315).

**Table 3:** Effect modification by prior treatment status: ΔP1NP and ΔTRACP-5b as predictors of the 24-month cumulative LS-BMD change.

Subgroup	n	ΔP1NP ρ	p-value	T1 vs. T3 BMD%	p
All patients	129	−0.375	<0.001	23.5% vs 12.3%	0.0003
Treatment-naïve	71	−0.359	0.002	28.7% vs 15.4%	0.001
Previously treated	58	−0.207	0.120	19.2% vs 12.4%	0.199
Denosumab sequential therapy	84	−0.354	0.001	25.8% vs 14.2%	0.012
Bisphosphonate sequential therapy	30	−0.350	0.058†	21.1% vs 13.9%	0.089

T1, highest ΔP1NP decline tertile; T3, lowest decline tertile. BMD% and median cumulative %YAM change. Interaction term (ΔP1NP × naïve/experienced): β=−0.072, p=0.024. †Likely underpowered (n=30).

## Discussion

This real-world cohort study demonstrated that on-treatment ΔP1NP, the change in P1NP from pre-treatment to approximately 1 month after completing romosozumab therapy, is an independent predictor of 2-year cumulative LS-BMD gain after sequential therapy, after adjustment for baseline BMD, prior treatment history, sequential therapy type, and 12-month BMD response. To our knowledge, this is the first study to demonstrate that the magnitude of P1NP suppression during romosozumab treatment provides independent prognostic information for long-term BMD outcomes, distinct from the information provided by baseline marker values or sequential therapy choice.

The biological rationale for this association is related to the dual mechanism of action of romosozumab. By inhibiting sclerostin, romosozumab activates osteoblasts (anabolic effect) and suppresses osteoclastogenesis (antiresorptive effect) (1). The early anabolic phase drives a transient P1NP increase, followed by a decline as the antiresorptive effect predominates, and TRACP-5b declines consistently from early treatment (11). One possible—though speculative—interpretation is that patients with greater net P1NP suppression at treatment completion may have a more pronounced antiresorptive response to romosozumab, potentially creating a lower bone remodeling environment that is more amenable to consolidation by subsequent antiresorptive therapy. However, the precise biological mechanism linking on-treatment P1NP dynamics to long-term post-sequential BMD remains to be established, and causal interpretation should be avoided at this stage of research.

A critical contextual finding from our previous work in this cohort (9) was that baseline P1NP as a continuous variable did not independently predict 12-month BMD (β=0.011, p=0.375). The present study shows that ΔP1NP (treatment-induced change) independently predicts 24-month BMD (β=−0.061, p=0.010). This contrast underscores that the dynamic pharmacodynamic response to romosozumab, captured by measuring BTMs at treatment completion, provides clinical information that is not available at baseline.

The ROC-derived optimal cutoff of ΔP1NP ≤−40.3 μg/L yielded good specificity (85.4%) for identifying good responders at 24 months of treatment. The AUC of 0.761 indicates moderate-to-good discriminative ability, which is clinically meaningful, given the number of confounders influencing long-term BMD outcomes. Importantly, absolute ΔP1NP outperformed the percentage change in all metrics (AUC, AIC, and standardized regression coefficient), suggesting that the absolute decline in P1NP—measured in the same units as the clinical reference range—is the more appropriate monitoring parameter in routine practice.

The significant interaction between ΔP1NP and prior treatment status (p=0.024) has practical implications. Treatment-naïve patients showed a robust ΔP1NP–BMD association (ρ=−0.359, T1 vs. T3 difference: 13.3 percentage points), whereas previously treated patients did not (ρ=−0.207, not significant). This likely reflects attenuated bone turnover in previously treated patients, particularly those on long-term bisphosphonates or prior denosumab, who present with lower baseline BTMs and smaller treatment-induced changes, reducing the dynamic range of ΔP1NP as a predictor. Clinicians monitoring BTMs during romosozumab treatment in previously treated patients should interpret ΔP1NP with caution, as its predictive value may be limited in this subgroup.

The consistency of the findings across the denosumab subgroup (ρ=−0.354, p=0.001, n=84) supports the robustness of the ΔP1NP–BMD relationship, independent of the specific sequential therapy chosen. The non-significant result in the bisphosphonate subgroup (p=0.058) likely reflects insufficient statistical power (n=30) rather than a true biological difference, which is consistent with the numerically similar correlation coefficient. Together with our prior demonstration that denosumab sequential therapy achieves greater 24-month BMD gains than bisphosphonates (13), the present findings suggest that ΔP1NP provides prognostic information about long-term BMD maintenance that is complementary to and independent of sequential therapy selection.

This study had several limitations. First, its single-center retrospective design limits generalizability, and unmeasured confounders (adherence, fall risk, nutritional status, and prior treatment duration) may influence outcomes. Second, BTM measurements were available at only two time points; serial assessments at 1, 3, 6, and 12 months would better characterize the P1NP trajectory and may improve the predictive accuracy. Third, the ΔP1NP cutoff of −40.3 μg/L requires prospective validation in an independent cohort study. Fourth, the bisphosphonate subgroup was underpowered (n=30), precluding definitive conclusions regarding the sequential therapy-specific effects. Fifth, the predominantly female Japanese population (96.9%) limits the generalizability of the results to men and non-Asian populations. Sixth, BMD was expressed as %YAM, a standard Japanese metric, which limits direct comparison with T-score-based international studies. Seventh, this study used BMD as the primary outcome, which is an established surrogate endpoint but does not directly reflect the fracture risk reduction. Whether the ΔP1NP-based prediction of BMD translates into clinically meaningful differences in fracture incidence remains to be demonstrated in prospective studies with fractures as the primary endpoint. Eighth, the ROC-derived cutoff of ΔP1NP ≤−40.3 μg/L was derived from this single cohort and has not been externally validated; the clinical implementation of this threshold should be deferred until validation in independent, prospective cohorts of sufficient size. Finally, the predominantly female Japanese population (96.9% female) and single-center retrospective design substantially limit the generalizability of these findings to male patients, non-Asian populations, and settings with different clinical practices or BTM assay platforms. These limitations should be explicitly acknowledged when applying our findings to future studies. Finally, PTH and serum phosphate levels were not systematically collected in this cohort and therefore could not be included in the biochemical characterization. Eleventh, secondary osteoporosis was not systematically excluded from the protocol, although clinical judgment was applied in patient selection.

## Conclusion

On-treatment ΔP1NP is an independent predictor of the 2-year cumulative LS-BMD after sequential therapy following romosozumab, with the greatest predictive value in treatment-naïve patients. A ΔP1NP ≤−40.3 μg/L at treatment completion identifies patients likely to achieve superior long-term BMD gains, with a specificity of 85.4%. These findings suggest that on-treatment BTM monitoring during romosozumab treatment may have clinical utility for long-term prognosis. However, given the single-center retrospective design, predominantly female Japanese population (96.9%), small bisphosphonate subgroup (n=30), and need for external validation of the ΔP1NP cutoff (≤−40.3 μg/L), prospective multicenter studies are warranted before clinical implementation of this threshold.

## Data Availability

The dataset supporting the conclusions of this article is not publicly available due to patient privacy regulations and the requirements of the institutional ethics approval (Shimokita Medical Center Association IRB, approval number: RO7-5). The data contain sensitive clinical information collected during routine medical care at a single center in Japan and are subject to the Act on the Protection of Personal Information (APPI, Japan). Data sharing is not permitted under the terms of the ethics approval. Requests to access de-identified summary data may be directed to the corresponding author (Ryo Nakano, MD; riemann.2.43112609@icloud.com) and will be considered on a case-by-case basis, subject to institutional approval and the execution of a data sharing agreement. Requests to access the datasets should be directed to Ryo Nakano, MD; riemann.2.43112609@icloud.com.
