# Supplementary material for: On-treatment change in bone turnover markers predicts 2-year bone mineral density after sequential therapy following romosozumab: a real-world cohort study
Source: Front Endocrinol (Lausanne). 2026 Jun 17;17:1858266. doi: 10.3389/fendo.2026.1858266 (PMC13318748; doi:10.3389/fendo.2026.1858266)
Supplement: Supplementary file 1 [file Table1.docx]

**Supplementary Table S1.** Baseline and post-treatment biochemical parameters in the primary analysis population (n=129).

| **Variable** | **n** | **Mean ± SD** | **Median** | **Range** |
| --- | --- | --- | --- | --- |
| Baseline |  |  |  |  |
| Corrected Ca (mg/dL) | 129 | 9.3 ± 0.8 | 9.3 | 2.0–10.8 |
| Serum albumin (g/dL) | 124 | 4.0 ± 0.4 | 4.1 | 2.8–4.7 |
| Total protein (g/dL) | 40† | 6.9 ± 0.6 | 7.0 | 5.3–8.1 |
| eGFR (mL/min/1.73m²) | 129 | 68.5 ± 18.6 | 69.0 | 26–142 |
| 25-OH Vitamin D (ng/mL) | 111 | 17.0 ± 7.4 | 16.5 | 4.6–55.6 |
| BMI (kg/m²) | 128 | 21.4 ± 4.0 | 21.6 | 10.2–33.3 |
| Post-treatment (approximately 1 month after 12th dose) |  |  |  |  |
| Corrected Ca (mg/dL) | 129 | 9.4 ± 0.5 | 9.4 | 7.8–12.5 |
| eGFR (mL/min/1.73m²) | 129 | 65.5 ± 16.2 | 66.0 | 25–112 |
| Not measured |  |  |  |  |
| Serum PTH | — | Not collected | — | — |
| Serum phosphate | — | Not collected | — | — |

*Data are mean ± SD, or median, as appropriate. eGFR, estimated glomerular filtration rate; Ca, calcium; BMI, body mass index; PTH, parathyroid hormone.*

*†Total protein was recorded in 40 patients (31.0%); this reflects documentation practice rather than selective measurement.*

**Supplementary Table S2.** Sensitivity analyses: ROC performance of ΔP1NP under alternative definitions of good response.

| **Definition of good response** | **Threshold** | **n (%) good responders** | **AUC (ΔP1NP)** | **Optimal cutoff (μg/L)** | **Sensitivity / Specificity** |
| --- | --- | --- | --- | --- | --- |
| ≥75th percentile (primary) | ≥24.6% cumulative LS-BMD | 33 (25.6%) | 0.761 | −40.3 | 0.545 / 0.854 |
| ≥50th percentile | ≥14.5% cumulative LS-BMD | 65 (50.4%) | 0.704 | −33.3 | 0.462 / 0.891 |
| ≥Least Significant Change | ≥3% cumulative LS-BMD | 119 (92.2%) | 0.767 | +7.7 | 0.697 / 0.800 |
| ≥15% (clinical improvement) | ≥15% cumulative LS-BMD | 63 (48.8%) | 0.689 | −33.3 | 0.460 / 0.879 |

*AUC values remained in the moderate-to-good range (0.689–0.767) across all definitions, supporting the robustness of the primary analysis. LS-BMD, lumbar spine bone mineral density; AUC, area under the receiver operating characteristic curve.*
